# Distilled Tar Water in the Treatment of Hemorrhages

**Published:** 1888-10

**Authors:** 


**Distilled Tar Water in the Treatment of Hemorrhages.**—(*Corneville Saint Marc.*) The author has administered distilled tar water in a large number of cases. The results of his observations are that the products constitute an excellent hemostatic, whose properties present great analogy to those of hamamelis Virginica. He concludes that tar water, prepared from wood tar, constitutes a medicine with incontestible tonic, astringent properties. Administered internally it arrests surely and rapid hemorrhages of the lungs of congestive origin, of the uterus and the kidneys. It offers the surest and most prompt means to arrest the hemorrhages of the first two periods of pulmonary tuberculosis. The dose is from 40 to 60 grammes in twenty-four hours. He has never had the least bad result to follow the use of this remedy.—*Revue de Theraputique.*

